# Long-term use of drugs used for knee osteoarthritis and their association with primary arthroplasty: a nested case-control study

**DOI:** 10.1186/s42836-026-00421-7

**Published:** 2026-08-07

**Authors:** Sara Rodríguez-Martín, Encarnación Fernández-Antón, Gabriela Alonso-Martínez, Miguel Gil, Antonio Rodríguez-Miguel, Nina Martínez, Josep Vergés, Francisco J. de Abajo

**Affiliations:** 1 Department of Biomedical Sciences (Pharmacology), School of Medicine and Health Sciences, University of Alcalá (IRYCIS), Alcalá de Henares, Madrid, 28805 Spain; 2 Division of Pharmacoepidemiology and Pharmacovigilance, Spanish Agency of Medicines and Medical Devices (AEMPS), Madrid, 28022 Spain; 3 Osteoarthritis Foundation International, Barcelona, 08006 Spain; 4 Clinical Pharmacology Unit, University Hospital Príncipe de Asturias, Alcalá de Henares, Madrid 28805 Spain

**Keywords:** Knee osteoarthritis, Knee arthroplasty, Non-steroidal anti-inflammatory drugs (NSAIDs), Symptomatic slow-acting drugs for osteoarthritis (SYSADOAs), Pharmacoepidemiology, Nested case-control study, Real-world data

## Abstract

**Background:**

Knee osteoarthritis is a highly prevalent condition that eventually ends up in arthroplasty at advanced stages. It is unknown whether the prolonged use of drugs for its symptomatic treatment, including non-steroidal anti-inflammatory drugs (NSAIDs), opioids, non-opioid analgesics, and symptomatic slow-acting drugs for osteoarthritis (SYSADOAs), has an impact on the risk of arthroplasty. The purpose of this study was to assess whether long-term use of different medications prescribed for the symptomatic treatment of knee osteoarthritis is associated with the risk of undergoing surgical joint replacement compared with short-term use.

**Design:**

A case-control study nested in a cohort of 264,014 patients with a recorded diagnosis of knee osteoarthritis was carried out in BIFAP, a Spanish primary healthcare database. From this cohort, we identified 15,892 cases of primary knee arthroplasty and matched them with up to 3 controls on age, sex, healthcare district, and index date using risk-set sampling. Through a conditional logistic regression, we computed the adjusted odds ratios (AOR) and 95% confidence intervals (95% CI) associated with durations of treatment 1–3, > 3–5, and > 5 years in comparison with < 1 year within the same pharmacological class. A test for trend was carried out.

**Results:**

A trend to an increased risk of arthroplasty was found as treatment duration of NSAIDs increases, while no such trend was observed with SYSADOAs or analgesics. Patients using NSAIDs for more than 5 years showed an AOR of 2.35 (2.10–2.63) as compared to < 1 year. Both males and females, and subjects younger and older than 70 years old, shared this pattern.

**Conclusions:**

This observational study shows that the prolonged use of NSAIDs in patients with knee osteoarthritis is associated with an increased risk of arthroplasty, while residual confounding by OA severity cannot be excluded. Results from SYSADOAs, opioids, and non-opioid analgesics are consistent with no association.

**Supplementary Information:**

The online version contains supplementary material available at 10.1186/s42836-026-00421-7.

## Background

Osteoarthritis (OA) is the most common chronic joint disorder in Western societies and a leading cause of disability in terms of years lived with disability worldwide [1, 2], and the knee is one of its most disabling locations of OA [3]. Age, female sex, obesity, prior joint trauma, and certain occupational exposures are well-established risk factors for knee OA [3–6]. In addition to non-pharmacological measures, there are several drugs available to reduce symptoms, especially pain [3], but when the degeneration of the articular cartilage reaches advanced stages, and the patients’ disability is high, it is common to offer patients surgical joint replacement (total or partial) with a prosthesis (arthroplasty). In a study conducted using a primary care database in Catalonia (Spain) with data from 2006–2015, the lifetime prevalence of total arthroplasty was estimated at 30% (95% CI: 25%–36%) in patients with knee OA [7]. It is estimated that in the USA, around 700,000 primary total knee replacements [8] are done annually. Although arthroplasty is effective in treating the symptoms of OA, it is usually performed in the final stage of the disease, after years of pain and loss of function. During this time, treatment includes the use of nonsteroidal anti-inflammatory drugs (NSAIDs), nonopioid analgesics (e.g., paracetamol [acetaminophen in the USA] and metamizole), opioid analgesics (alone or in combination with other analgesics), symptomatic slow-acting drugs for osteoarthritis (SYSADOAs, e.g., chondroitin sulphate) and intra-articular injections of corticosteroids or hyaluronic acid. The chronic use of all of these drugs has been controversial for different reasons: (1) NSAIDs, for their adverse gastrointestinal and cardiovascular effects [9]; (2) paracetamol, for its limited efficacy, especially when OA is advanced [10]; (3) opioids, for their addictive potential [11] and (4) SYSADOAs, for their controversial efficacy results [12–15], which has led to contradictory recommendations for and against their use in the main published clinical guidelines for the OA treatment [16]. Clinical guidelines also advocate the use of topical NSAIDs for knee OA [17], although their efficacy has been questioned for several years. Of all these, pharmaceutical-grade SYSADOAs have been postulated to have beneficial effects on disease progression; specifically, chondroitin sulfate (CS) has been reported to reduce knee cartilage volume loss [18] and minimal joint space narrowing [19]. Therefore, it may be conceivable that the long-term use of SYSADOAs is associated with a lower risk of arthroplasty. On the contrary, NSAIDs have been reported to interfere with the repair mechanisms, which, in the long term, might accelerate the progression of OA [20] and increase the risk of arthroplasty.

The main objective of the present study was to assess the association between long-term use of different drugs prescribed for the symptomatic treatment of knee OA and the risk of surgical joint replacement as compared to their short-term use.

## Methods

### Data source

For the present study, we used BIFAP (Base de datos para la Investigación Farmacoepidemiológica en el Ámbito Público), a Spanish database that contains pseudonymized electronic medical records from primary care practitioners [21]. The population included in BIFAP reflects the distribution of the Spanish population by sex and age. This database has been validated through numerous pharmacoepidemiological studies in different areas [21–25]. About half of the BIFAP population belongs to seven Autonomous Communities which, in addition to primary healthcare records, provide the codes of the hospital diagnosis discharge database (called “Conjunto Mínimo Básico de Datos”; CMBD). Thus, in order to assure the accuracy of the arthroplasty records (including dates), we restricted the study population to those seven communities with a CMBD link (from herewith BIFAP-CMBD).

### Study design

We built a cohort composed of patients (males or females) registered in BIFAP, aged 40 to 89 years, with at least 1 year of follow-up with their primary care physician and a first recorded diagnosis of knee OA. The first day subjects fulfilled the criteria of age, and 1-year registry was considered the “start date”. Patients were excluded if, before the start date, they had a record of cancer, hip, knee, or femur fracture, a major traumatism requiring hospitalization, or any inflammatory arthropathy (excepting gout). From this BIFAP OA knee cohort, we kept only those patients from autonomous communities that provided data from the CMBD database (hospital diagnosis) and had at least one recorded prescription for any of the drugs of interest (see below) over the study period (2005–2023). Additionally, we excluded “prevalent regular users” defined as those who had consecutive prescriptions for a duration of 3 months or longer in the last year before the start date, to ensure that patients included in the study cohort were “new users”. Since then, follow-up was carried out until the occurrence of any of the following events: a primary knee replacement, turning 90 years old, death, or end of study period (31 December 2023).

The date on which the event of interest occurs (the so-called index date) is crucial in any epidemiological study in order to only consider exposures that occurred before that date and avoid reverse causation. With regard to arthroplasty, we were able to ascertain with reasonable certainty the date on which the surgery was performed using the hospital discharge database. However, this is a scheduled surgery and according to the Spanish Ministry of Health, the average waiting time for knee arthroplasty is about 120 days [26]. As the exposures of interest for this study are those occurring before the decision to operate was made and this is not systematically recorded in BIFAP, we approximate it by moving back 180 days from the recorded date of surgery. A timeline diagram is shown in supplementary Fig. S1. Sensitivity analyses were performed with 120 and 365 lag times.

Nested in the study cohort, we performed a case–control study, with cases being patients with an incident primary knee arthroplasty and up to 3 controls per case individually matched with cases on age (± 2 years), sex, healthcare district, and index date. Healthcare district was taken as a proxy for socioeconomic status. Controls were randomly sampled from the respective source population following a risk-set sampling, an incidence-based sampling method which assures that the odds ratios (OR) calculated in the case-control study are unbiased estimates of rate ratios of the underlying cohort [27], additionally avoiding immortal time bias [28], a relevant issue when duration of treatments is to be analyzed. Controls were patients still at risk of arthroplasty who had not undergone arthroplasty by the matched index date, and who could later become cases.

### Definition of drug exposure

The pharmacological classes included in the present study were: SYSADOAs (including glucosamine [sulfate or hydrochloride] and CS; ATC codes: M01AX05, M01AX25; diacerein was not considered), systemic NSAIDs (ATC codes: M01AAxx, M01ABxx, M01ACxx, M01AExx, M01AGxx, M01AHxx, M01AX01, M01AX02 and M01AX24), non-opioid analgesics (including paracetamol and metamizole; ATC codes: N02BE01 and N02BB02), tramadol in fixed-dose combination with paracetamol (N02AJ13) and the rest of opioid analgesics, including combinations (ATC codes: N02Axxx and N07BCxx, excluding N02AJ13). Patients were classified as “users” of the specific drug of interest when they presented at least one prescription beginning after the start date and before the index date. Broadly, drugs were grouped by pharmacological classes as described before; for some of them, we also performed the analysis by subgroups (e.g., COX-2 selective or non-selective for NSAIDs) or active principle (e.g., CS and glucosamine, for SYSADOAs).

Treatment duration among users was calculated by adding consecutive prescriptions (accepting a gap of up to 90 days between the end of a prescription and the beginning of the next), and then clustered into 4 groups: < 1 year, 1–3 years, > 3–5 years and > 5 years (when the numbers were small, the last two categories were merged, > 3 years).

### Covariables at index date

We assessed the association of arthroplasty with the following factors at index date: obesity (recorded as such), diabetes (recorded as such and/or use of glucose-lowering drugs), ischemic stroke (including non-specified stroke and transient ischemic attack), hemorrhagic stroke, heart failure, angina pectoris (recorded as such and/or use of nitrates), myocardial infarction, peripheral arterial disease, hypertension, dyslipidemia (recorded as such and/or use of lipid-lowering drugs), atrial fibrillation, venous thromboembolic disease, chronic obstructive pulmonary disease, asthma, asymptomatic hyperuricemia, gout, chronic kidney disease (recorded as such), cirrhosis, chronic hepatitis, depression, anxiety, Alzheimer disease, non-specified dementia and Parkinson disease. None of these variables appear to be intermediate variables between OA drug treatment and surgical joint replacement, so all were considered potential confounding factors. Furthermore, we considered the number of visits to a primary care physician during the year prior to the index date as a proxy for comorbidity and fragility. Unfortunately, no score of OA severity was systematically available in BIFAP (e.g., Kellgren–Lawrence scale).

### Statistical analysis

For qualitative variables, the absolute frequency and percentages of each category were calculated, while quantitative variables were summarized with the mean and the standard deviation. The association between drug exposure and incident knee arthroplasty was evaluated by calculating the ORs and their 95% confidence intervals (95% CI) through a conditional logistic regression, adjusting for the covariates mentioned above.

**Fig. 1:**
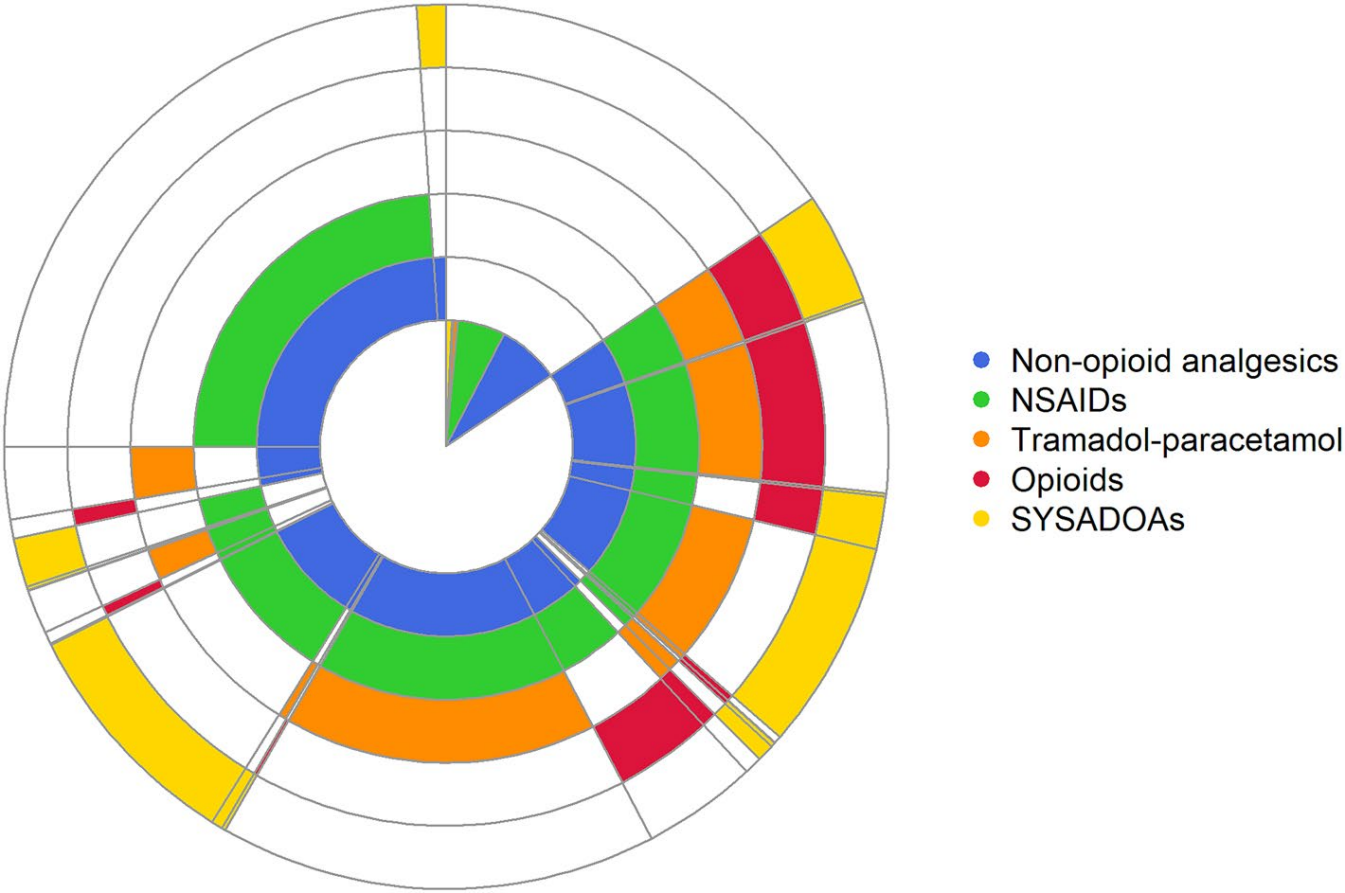
Different drugs used for symptomatic treatment of knee OA, either concomitantly or sequentially. The colors of the different concentric rings indicate therapeutic categories: non-opioid analgesics (paracetamol or metamizole) (blue), NSAIDs (green), tramadol and paracetamol in fixed-dose combination (orange), opioids (red), and SYSADOAs (yellow), in descending order of frequency. Overlapping rings indicate that most patients use different therapeutic classes over the study period, either concomitantly or sequentially. The central isolated portion of the chart represents patients who used only one therapeutic category throughout the follow-up period, without concurrent or sequential use of other therapeutic categories; it is noteworthy they are actually few of them

Most patients with knee OA were prescribed multiple drugs for symptomatic treatment over the study period, either sequentially or concomitantly (see Fig. 1), making it difficult to form comparable groups in an observational setting. Additionally, patients with a more severe OA are likely to be prescribed drugs with stronger analgesic effects, which may introduce a channeling bias. To circumvent all these problems, and considering that our main objective was to examine the association between treatment duration of specific drugs and the risk of joint replacement, we focused on the comparison between different long-term durations (i.e., 1–3 years, > 3–5 years and > 5 years) and short-term use (< 1 year), within each specific drug or pharmacological class; e.g., comparing long-term NSAIDs users with short-term NSAIDs users. For the same reason, we ruled out the possibility of employing a non-user group as the reference since patients with no record of prescription drugs for OA symptoms are presumably in an early stage of the disease and are therefore less likely to undergo joint replacement. This would have resulted in a spurious positive association between all drugs examined and the risk of joint replacement surgery. We included all pharmacological groups of interest at a time in order to adjust for multiple use (e.g., the association of the use of SYSADOAs with arthroplasty was adjusted for the concomitant or sequential use of NSAIDs, non-opioid analgesics, and opioids).

A potential interaction with age (stratified as under 70 and 70 years or older) and sex was examined through a stratified analysis using the Altman and Bland test to assess statistical significance [29]. Additionally, three sensitivity analyses were performed: (1) adjusting for the potential confounding factors ascertained at start date of the study cohort rather than at index date; (2) including in the model the follow-up time since start date to index date, to adjust for the potential influence that a different follow-up time between cases and controls may have on the results associated with treatment duration; and (3) moving back the index date 120 and 365 days before recorded surgery date.

Analyses were performed with STATA version 19/SE software (StataCorp, College Station, TX, USA).

## Results

**Fig. 2:**
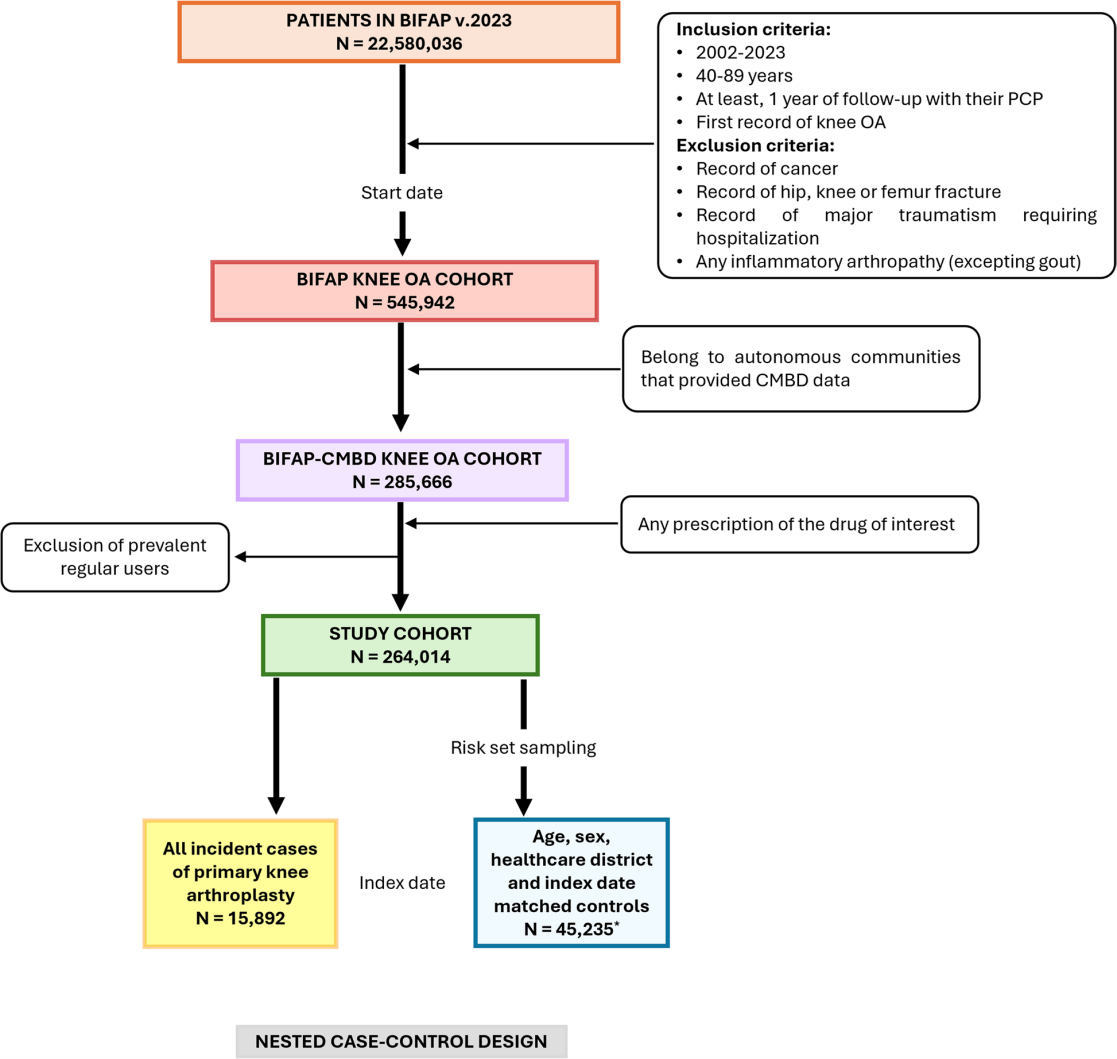
Flowchart of patient selection. *Abbreviations*: BIFAP: Base de datos para la Investigación Farmacoepidemiológica en el Ámbito Público; CMBD: Conjunto Mínimo Básico de Datos (a hospital discharge database); OA: osteoarthritis; PCP: primary care physician. *Note: 13,742 cases were matched with 3 controls (86.5%), 1,859 with 2 controls (11.7%), and 291 with just 1 control (1.8%)

A cohort of 545,942 patients with a recorded diagnosis of knee OA and fulfilling the inclusion criteria was selected from BIFAP. Of them, 264,014 patients belonged to the autonomous communities that provided CMBD codes and were new users of the drugs of interest. From this source population, a total of 15,892 incident cases of primary knee arthroplasty were identified and matched with 45,235 incidence-based sampled controls (Fig. 2). The crude incidence rate of knee arthroplasty in the study cohort was 69.3 per 10,000 person-years.

Characteristics of cases and controls at index date are shown in Table 1. The mean age at the time of joint replacement was 70.5 years (± 8.4), and women outnumbered men (62.5% vs. 37.5%). The mean follow-up time was practically identical in cases and controls. Compared with controls, cases had a higher prevalence of cardiovascular risk factors, such as obesity, diabetes, dyslipidemia, hypertension, and hyperuricemia, but a lower prevalence of established cardiovascular diseases (peripheral artery disease, heart failure, angina pectoris, myocardial infarction, and stroke) and severe chronic conditions such as Alzheimer´s disease and dementia. Depression, anxiety, asthma, and Parkinson disease were also more prevalent among cases.

**Table 1:** Characteristics of primary knee arthroplasty cases and matched controls at index date

	**Cases**	**Controls**	**Crude OR (95% CI)**
	**N = 15,892**	**N = 45,235**	
Mean age (SD)	70.5 (± 8.4)	70.7 (± 8.4)	Matched
Women (%)	9939 (62.5)	28562 (63.1)	Matched
Mean follow-up time, in years (SD)	6.6 (± 3.0)	6.7 (± 2.3)	0.99 (0.97–1.01)
Number of visits (last 12 months)			
Up to 5	6133 (38.6)	18243 (40.3)	1 (ref.)
6–15	6765 (42.6)	18723 (41.4)	1.07 (1.03–1.12)
16–24	1523 (9.6)	4125 (9.1)	1.10 (1.02–1.18)
25 +	1471 (9.3)	4144 (9.2)	1.05 (0.98–1.12)
Obesity (%)	1312 (8.3)	2404 (5.3)	1.65 (1.54–1.77)
Diabetes^†^ (%)	2669 (16.8)	5046 (11.2)	1.62 (1.54–1.71)
Dyslipidemia^‡^ (%)	6244 (39.3)	9661 (21.4)	2.45 (2.35–2.55)
Peripheral artery disease (%)	205 (1.3)	685 (1.5)	0.86 (0.74–1.01)
Hypertension (%)	8590 (54.1)	12325 (27.3)	3.41 (3.27–3.55)
Heart failure (%)	328 (2.1)	1207 (2.7)	0.78 (0.69–0.88)
Atrial fibrillation (%)	861 (5.4)	2233 (4.9)	1.12 (1.03–1.21)
Angina pectoris^§^ (%)	91 (0.57)	272 (0.60)	0.97 (0.77–1.24)
Myocardial infarction (%)	76 (0.48)	271 (0.60)	0.82 (0.64–1.06)
Ischemic stroke^||^ (%)	222 (1.4)	721 (1.6)	0.89 (0.77–1.04)
Hemorrhagic stroke (%)	7 (0.04)	58 (0.13)	0.35 (0.16–0.76)
Venous thromboembolic disease (%)	160 (1.0)	460 (1.0)	1.01 (0.84–1.21)
Asymptomatic hyperuricemia (%)	673 (4.2)	1221 (2.7)	1.61 (1.46–1.78)
Gout (%)	153 (0.96)	406 (0.90)	1.08 (0.90–1.31)
Chronic obstructive pulmonary disease (%)	558 (3.5)	1433 (3.2)	1.12 (1.01–1.23)
Asthma (%)	851 (5.4)	1409 (3.1)	1.78 (1.63–1.94)
Chronic hepatitis (%)	54 (0.34)	133 (0.29)	1.13 (0.82–1.56)
Cirrhosis (%)	19 (0.12)	53 (0.12)	1.02 (0.60–1.72)
Chronic kidney disease (%)	519 (3.3)	1450 (3.2)	1.04 (0.93–1.15)
Depression (%)	825 (5.2)	1645 (3.6)	1.49 (1.37–1.63)
Anxiety (%)	224 (1.4)	521 (1.2)	1.25 (1.07–1.47)
Alzheimer disease (%)	28 (0.18)	295 (0.65)	0.27 (0.19–0.40)
Dementia (%)	13 (0.08)	83 (0.18)	0.45 (0.25–0.80)
Parkinson disease (%)	70 (0.44)	120 (0.27)	1.68 (1.24–2.25)

^†^Recorded as such and/or when patients were using glucose-lowering drugs

^‡^Recorded as such and/or when patients were using lipid-lowering drugs

^§^Recorded as such and/or when patients were using nitrates

^||^Including non-specified stroke and transient ischemic attacks

### Use of NSAIDs and knee arthroplasty

**Fig. 3:**
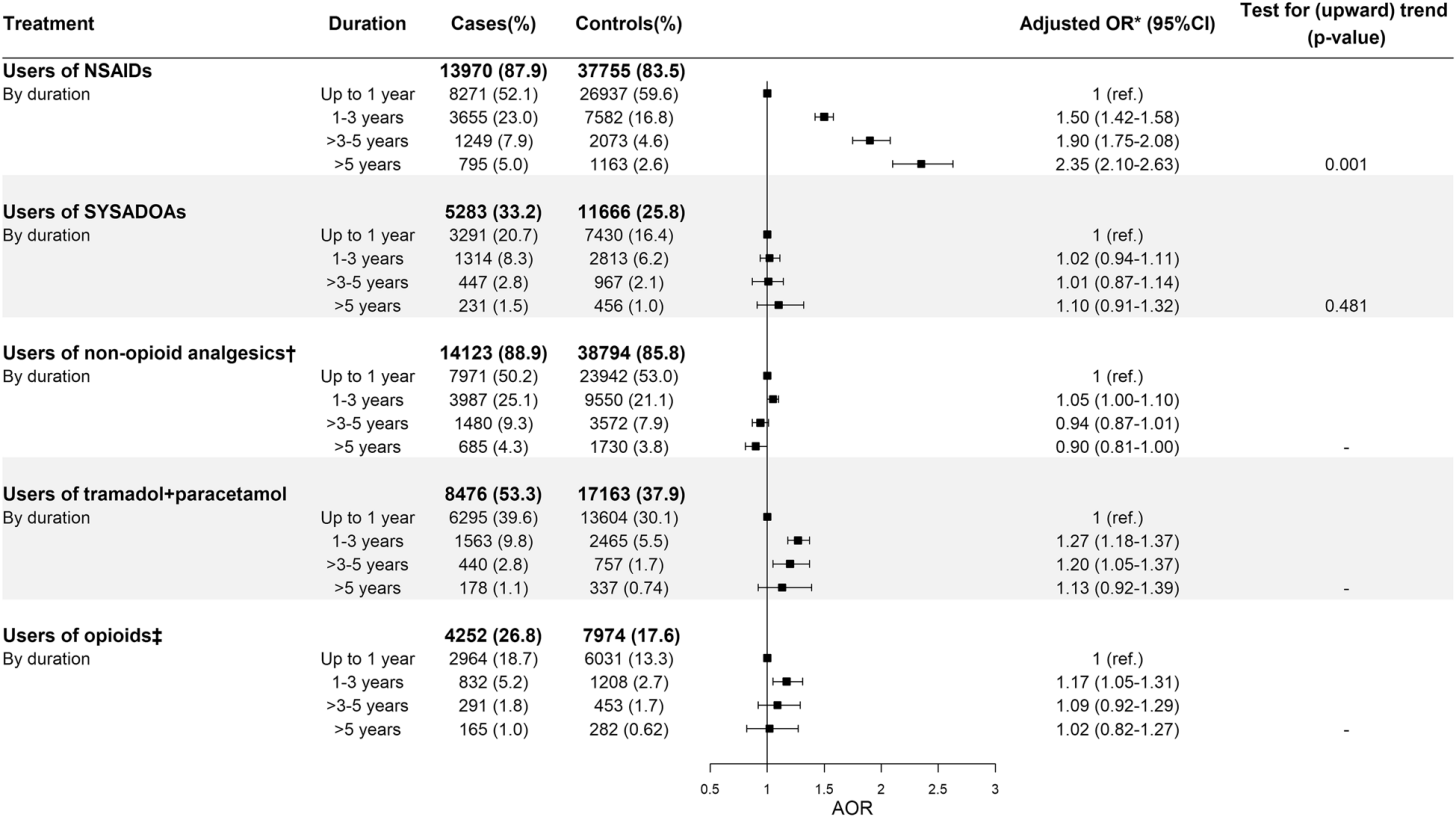
Risk of knee arthroplasty associated with treatment duration of drugs used for osteoarthritis. *Abbreviations*: NSAIDs: non-steroidal anti-inflammatory drugs; OR: odds ratio; SYSADOAs: symptomatic slow-acting drugs for osteoarthritis. † Includes paracetamol or metamizole; ^‡^ Includes all opioids except tramadol in fixed-dose combination with paracetamol; * All different pharmacological groups (NSAIDs, SYSADOAs, non-opioid analgesics, tramadol in fixed-dose combination with paracetamol, and opioids) were included at a time and additionally adjusted for age, sex, number of visits to primary care physician during the year prior to index date, obesity, diabetes (recorded as such and/or use of glucose-lowering drugs), dyslipidemia (recorded as such and/or use of lipid-lowering drugs), peripheral artery disease, hypertension, heart failure, atrial fibrillation, angina pectoris (recorded as such and/or use of nitrates), myocardial infarction, ischemic stroke (including non-specified stroke and transient ischemic attack), hemorrhagic stroke, chronic obstructive pulmonary disease, asthma, hyperuricemia, gout, thromboembolic disease, chronic hepatitis, cirrhosis, chronic kidney disease, depression, anxiety, Alzheimer’s disease, dementia, and Parkinson disease

A total of 13,970 cases (87.9%) and 37,755 controls (83.5%) had ever used NSAIDs over the study period. We found a strong and statistically significant trend (*p* = 0.001) to an increased risk of arthroplasty as duration of treatment increases (Fig. 3). The adjusted OR of a duration > 5 years vs. a duration < 1 year was 2.35 (95% CI 2.10–2.63). The adjustment for the potential confounding factors at start date, instead of index date, did not materially change the results, nor did the adjustment for follow-up time (supplementary Tables S1 and S2). When NSAIDs were classified by COX-2 selectivity, we found a similar significant upward trend among coxibs and among non-selective NSAIDs (Table 2). Using 120 days or 365 days as the lag time instead of 180 days did not materially change the results (supplementary Table S3).

**Table 2:** Association of knee arthroplasty with NSAID use and treatment duration by COX-2 selectivity

	**Cases (%)**	**Controls (%)**	**Crude OR**	Adjusted OR*
	**N = 15,892**	**N = 45,235**	**(95% CI)**	**(95% CI)**
**Users of coxibs**	7336 (46.2)	13691 (30.3)		
**By duration:**				
< 1 year	5540 (34.9)	11564 (25.6)	1 (ref)	1 (ref)
1–3 years	1329 (8.4)	1658 (3.7)	1.77 (1.64–1.92)	1.53 (1.41–1.68)
> 3–5 years	313 (2.0)	321 (0.71)	2.22 (1.89–2.61)	2.07 (1.73–2.46)
> 5 years	154 (0.97)	148 (0.33)	2.46 (1.95–3.09)	2.39 (1.86–3.07)
				*p* for trend = 0.001
**Users of non-selective NSAIDs**	12998 (81.8)	35,954 (79.5)		
**By duration:**				
< 1 year	8366 (52.6)	26642 (58.9)	1 (ref)	1 (ref)
1–3 years	2994 (18.8)	6546 (14.5)	1.59 (1.51–1.67)	1.39 (1.31–1.47)
> 3–5 years	991 (6.2)	1750 (3.9)	2.11 (1.94–2.30)	1.71 (1.56–1.88)
> 5 years	647 (4.1)	1016 (2.3)	2.57 (2.30–2.86)	2.02 (1.79–2.28)
				*p* for trend = 0.001

*Abbreviations: CI* confidence interval, *NSAIDs* non-steroidal anti-inflammatory drugs, *OR* odds ratio

^*^Adjusted for factors shown in footnote of Fig. 3

### Use of SYSADOAs and knee arthroplasty

A total of 5,283 knee arthroplasty cases (33.2%) and 11,666 controls (25.8%) had ever used SYSADOAs over the study period. No upward trend (*p* = 0.481) was observed with treatment duration (Fig. 3; adjusted OR of > 5 years vs. < 1 year = 1.10; 95% CI 0.91–1.32). Results of SYSADOAs were mainly driven by CS (adjusted OR of > 5 years vs. < 1 year = 1.14; 95% CI 0.93–1.40), while glucosamine showed a non-significant downward trend (*p* = 0.267) (adjusted OR of > 5 years vs. < 1 year = 0.74; 95% CI 0.44–1.27) (Table 3). In the sensitivity analyses, the adjustment for the potential confounding factors at start date rather than at index date, or the adjustment for the follow-up time, did not materially change the results (supplementary Tables S4 and S5). The use of a lag time of 120 or 365 days instead of 180 did not change the results either (supplementary Table S6).

**Table 3:** Association of knee arthroplasty with SYSADOA use by active principle and treatment duration

	**Cases**	**Controls**	**Crude OR**	Adjusted OR*
	**N = 15,892**	**N = 45,235**	**(95% CI)**	**(95% CI)**
**Use of CS**	4810 (30.1)	10401 (23.0)		
**By duration:**				
< 1 year	3075 (19.4)	6750 (14.9)	1 (ref)	1 (ref)
1–3 years	1151 (7.2)	2464 (5.5)	1.05 (0.97–1.14)	0.99 (0.91–1.09)
> 3–5 years	393 (2.5)	839 (1.9)	1.08 (0.95–1.23)	0.97 (0.84–1.12)
> 5 years	191 (1.2)	348 (0.77)	1.30 (1.08–1.56)	1.14 (0.93–1.40)
				*p* for upward trend = 0.506
**Use of glucosamine**	1043 (6.6)	2366 (5.2)		
**By duration:**				
< 1 year	737 (4.6)	1680 (3.7)	1 (ref)	1 (ref)
1–3 years	225 (1.4)	477 (1.1)	1.09 (0.91–1.30)	1.08 (0.89–1.32)
> 3–5 years	59 (0.37)	139 (0.31)	0.99 (0.72–1.36)	1.02 (0.72–1.44)
> 5 years	22 (0.14)	70 (0.15)	0.75 (0.46–1.21)	0.74 (0.44–1.27)
				*p* for downward trend = 0.267

*Abbreviations: CI* confidence interval, *CS* chondroitin sulfate, *OR* odds ratio

^*^Adjusted for factors shown in footnote of Fig. 3

### Use of analgesics and knee arthroplasty

A total of 14,123 cases (88.9%) and 38,794 controls (85.8%) had ever used paracetamol or metamizole. No upward trend was observed with treatment duration (Fig. 3; see Table S7 for separate results by active principle). A total of 8,476 cases (53.3%) and 17,163 controls (37.9%) had ever used paracetamol and tramadol in fixed-dose combinations, and 4,252 cases (26.8%) and 7,974 (17.6%) controls used opioids (including tramadol as a single-drug medicinal product). No upward trend with duration of treatment was found with any of them (Fig. 3). Sensitivity analyses using 120 or 365 days as the lag time did not materially change the results (supplementary Tables S8–S10).

### Potential effect modification by sex or age

**Fig. 4:**
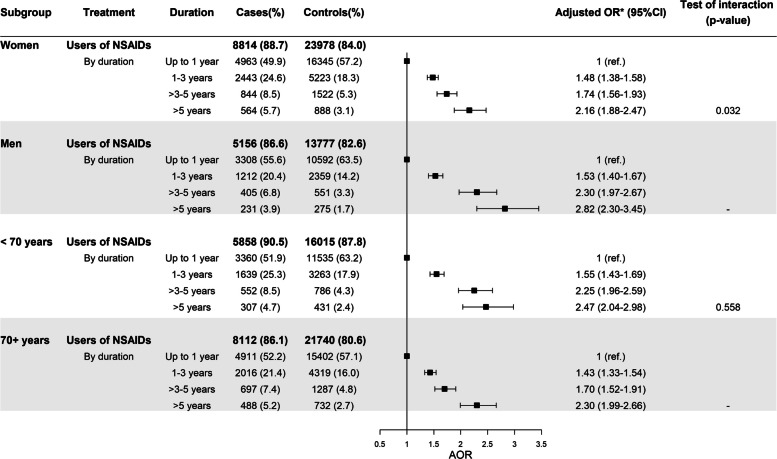
Assessment of effect modification by sex and age on NSAID-associated arthroplasty risk. Potential effect modification by sex and age group (< 70 and ≥ 70 years old) on the association of primary knee arthroplasty with the use of NSAIDs by duration of treatment. The test of interaction was done by comparing the adjusted OR of duration of use > 5 years vs. < 1 year in women with that of men, and the adjusted OR > 5 years vs. < 1 year observed in patients younger than 70 years old with that of patients aged 70 years or older. *Abbreviations*: CI: confidence interval; NSAIDs: non-steroidal anti-inflammatory drugs; OR: odds ratio. *Adjusted for factors shown in the footnote of Fig. 3

**Fig. 5:**
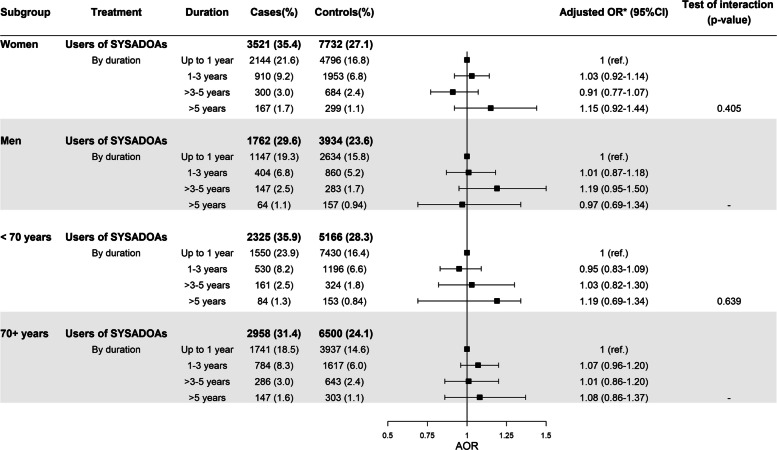
Assessment of effect modification by sex and age on SYSADOA-associated arthroplasty risk. Potential modification effect by sex and age group (< 70 and ≥ 70 years old) on the association of primary knee arthroplasty with the use of SYSADOAs by duration of treatment. The test of interaction was done comparing the adjusted OR of duration of use > 5 years vs. < 1 year in women with that of men, and the adjusted OR > 5 years vs. < 1 year observed in patients younger than 70 years old with that of patients aged 70 years or older. *Abbreviations*: CI: confidence interval; OR: odds ratio; SYSADOAs: symptomatic slow-acting drugs for osteoarthritis. *Adjusted for factors shown in the footnote of Fig. 3

Among users of NSAIDs, both women and men, and younger and older than 70 years of age presented a significant upward trend with increased duration of treatment (Fig. 4). Comparing AORs of NSAID duration > 5 years vs. < 1 year, significant differences were found between men and women (test of interaction, *p* = 0.032), but not between younger and older than 70 years old (test of interaction, *p* = 0.558). Among SYSADOAs, we did not observe a trend in any subgroup of sex or age, nor did we find an interaction between duration of treatment and these variables (Fig. 5).

For the rest of the drugs, the results by sex and age groups are shown in the supplementary material (Figs. S2, S3 and S4). No upward trend was observed with any pharmacological group in any subgroup of patients. Nevertheless, men and those younger than 70 years old showed a non-significant downward trend with treatment duration of non-opioid analgesics and fixed dose combinations of tramadol + paracetamol, respectively.

## Discussion

The main findings of the present study were as follows: (1) NSAIDs were the most commonly prescribed drugs in patients with knee OA (83% control patients with prescriptions over the study period and 24% with a continuous use over 1 year), while SYSADOAs were the drugs with the lowest prescription rate (26% control patients with prescriptions and 9% with a continuous use over 1 year); (2) the use of NSAIDs was associated with a steady trend to an increased risk of joint replacement as duration of treatment increases, so that when continuous use extended for more than 5 years, the associated risk of arthroplasty was more than double the one shown with short-term use (< 1 year); (3) the use of SYSADOAs was not associated with an upward or downward trend of an increased risk of arthroplasty as treatment duration increases; and (4) no upward trend was found with the prolonged use of either non-opioid or opioid analgesics.

Patients who underwent knee arthroplasty had a mean age of 71 years, a few years higher than the one reported in other countries (mid-60 s) [8], and women outnumbered men in accordance with the known epidemiology of knee OA [3]. Patients submitted to arthroplasty presented a higher prevalence of cardiovascular risk factors but a lower prevalence of established vascular diseases, as well as other serious diseases. These findings are compatible with the widely accepted idea that OA, in particular when it is in such an advanced stage as to require joint replacement, is associated with several cardiovascular risk factors, which may be the result of a chronic low-grade systemic inflammation [30]. However, once patients develop serious comorbid conditions that increase surgical risk, they may no longer be considered suitable candidates for joint replacement.

The most remarkable finding of our study is that the long-term use of NSAIDs was positively associated with a high and steady increased risk of arthroplasty. Such a pattern is observed in both males and females and in patients younger or older than 70 years old. Many studies have reported that NSAIDs inhibit the synthesis of proteoglycans and glycosaminoglycans [31–33] and, through the inhibition of prostaglandin E2 synthesis, impair the growth and differentiation of chondrocytes [34], thereby contributing to cartilage degeneration and OA progression. Also, clinical studies have long shown that NSAID use is associated with radiological progression of hip [35] and knee OA [36] and, specifically, a higher risk of surgical joint replacement [37–39]. Altogether, these findings provide biological plausibility for the observed association; however, our data do not directly prove that NSAIDs have a deleterious pharmacological effect on joints, and other explanations are possible: (1) the analgesic effect of NSAIDs may cause that patients with knee OA increase joint loading which, at long-term, has been hypothesized to accelerate the progression of the disease [40]; (2) long-term NSAID use may be associated with persistent symptoms, making these patients more likely to be selected by orthopedic surgeons for surgical joint replacement; and (3) patients who can continue long-term NSAID therapy may represent a selected group with fewer contraindications and potentially greater eligibility for surgery. Nevertheless, the fact that we did not find a similar upward and steady trend with the long-term use of other analgesics does not support these other explanations, although their prescription, particularly that of opioids, may be influenced by additional clinical, safety-related, and social factors. It is important to emphasize that in our study, NSAIDs were the most widely prescribed drugs in patients with knee OA and that around a quarter use them long-term (> 1 year), which deserves careful consideration in light of the observed association, in addition to their well-known gastrointestinal, cardiovascular and renal risks [9].

One of the main hypotheses tested in this study was whether long-term use of SYSADOAs was associated with a reduced risk of knee surgical replacement. Several experimental and clinical findings have supported this idea. For instance, CS has been reported to exert anti-inflammatory and anti-catabolic effects on cartilage metabolism, including the inhibition of metalloproteinases and inflammatory mediators involved in cartilage degradation [41, 42]. Moreover, some randomized clinical trials and imaging studies have reported that pharmaceutical-grade CS may reduce cartilage volume loss and slow joint space narrowing in knee OA, suggesting a potential structure-modifying effect [18, 43, 44]. Our results, obtained in a real-world setting, however, do not support a long-term reduced risk of knee arthroplasty associated with SYSADOA use and are rather compatible with a neutral effect. Perhaps, the effect observed in experimental conditions does not translate into a real-world setting due to several factors, such as the lack of adherence in the long term (only 12% of SYSADOA users presented a duration of 3 years or longer among controls) or the frequent concomitant or sequential use of NSAIDs (90% of SYSADOA users also used NSAIDs sequentially or concomitantly). SYSADOAs have been shown in randomized clinical trials to have a sustainable effect on pain in patients with knee OA, comparable to NSAIDs [14, 45], and with a much better gastrointestinal [46] and cardiovascular profiles [24, 47], which suggest that these drugs may still have a place in the symptomatic treatment of these patients, as supported by several guidelines [48, 49].

Almost 80% of subjects with knee OA had a prescription of paracetamol, and 37% of them used it for periods longer than 1 year. The association with arthroplasty was around the null value at different treatment durations, results compatible with a neutral effect on the long-term risk of joint replacement. Opioids were prescribed in around 56% of patients with knee OA among controls, although most of them for short-term use (around 8% used paracetamol combined with tramadol for 1 year or longer and around 5% used tramadol in higher doses as a single-drug or other strong opioids for such long periods). No upward trend was found between prolonged opioid use and risk of knee arthroplasty, although these patients are likely to be in an advanced stage of knee OA.

The strengths of the present study are as follows: (1) although the study was retrospective, the physicians collected patient data prospectively, including patient history, and a complete record of prescriptions filled; (2) the sample size of the study was large and allowed us to estimate risks with reasonable precision; (3) arthroplasty cases included in the study were only those recorded from the database of hospital discharge diagnosis, so a misclassification of the outcome is very unlikely; (4) controls were randomly extracted from the underlying cohort to make sure they represent the population exposure, this way, avoiding a selection bias; and (5) we excluded regular users of the drugs of interest before the start date, which prevented the introduction of a prevalent user bias [50] and allowed for an accurate assessment of treatment duration.

The present study has, however, several limitations. First, despite our efforts to control for confounding factors, a residual confounding due to unknown or unmeasured factors may be present; for instance, primary healthcare physicians do not systematically record the severity of knee OA (such as Kellgren–Lawrence scale), nor did they accurately record pain scores, BMI values, function, alignment, activity level, disease duration, orthopedic referral, or injection therapy, all of them predictors of joint replacement, and thus this information is lacking in BIFAP, making impossible the adjustment for these factors. Then, if NSAIDs were more prescribed than other drugs to patients with severe disease, it would be expected that an increased risk of arthroplasty would be associated with their use as compared to other drugs. To minimize this potential bias, we avoided cross-comparisons between different drugs, and just compared long-term with short-term use within each specific drug or pharmacological class. Second, all prescriptions are filled through the computer and then completely recorded, but treatment adherence by patients cannot be assured. Third, BIFAP only systematically includes prescription drugs; therefore, as some NSAIDs and paracetamol at low doses are available over the counter (OTC), the patients who use them were likely misclassified either as non-users (when they may actually be users of OTC NSAIDs) or short-term users (when they may actually be long-term users of OTC NSAIDs). Taking into account that short-term use is the category of reference in our analysis, one could conceive that a misclassification of long-term users (of OTC NSAIDs) as short-term users may have had an impact on our results; however, we consider this misclassification of the exposure would not explain the positive association and trend found with long-term use of NSAIDs for the following reasons: (1) it is likely that such misclassification is non-differential with respect to the case status (affecting equally both cases and controls); if this is so, the comparison between long-term use and short-term use would yield to a dilution of the OR, as the category of short-term users would be contaminated with long-term OTC NSAIDs users, meaning that the true OR would be likely stronger (not weaker) than the one estimated; and (2) it is expected that OTC paracetamol would also be affected, at least, in the same manner as OTC NSAIDs, but we do not see the same positive trend we observed with NSAIDs. On this matter, it is important to emphasize that CS and glucosamine included in this study are prescription-only medicines funded by the National Health System and, most probably, very different from the supplement-grade products available in many countries; therefore, our results with SYSADOAs cannot be generalized. Fourth, we did not include intra-articular therapies (steroids, hyaluronic acid) because they are usually administered in specialized settings and there is no systematic recording in BIFAP. Finally, topical NSAIDs were not included in this study as many are delivered over the counter.

In conclusion, the results of the present research do not support the hypothesis of a duration-dependent protective effect of SYSADOAs on arthroplasty in patients with knee OA; rather, they are more compatible with a neutral effect. The steady trend to an increased risk of arthroplasty associated with the long-term use of NSAIDs in patients with knee OA, as compared to their short-term use, is an important finding that may have implications for the long-term treatment of OA, although residual confounding by OA severity cannot be excluded.

## Supplementary Information

Supplementary Material 1.

## Data Availability

The data supporting the findings of this study are available from BIFAP, which is owned by the Spanish Agency for Medicines and Medical Devices (AEMPS). The investigators were granted permission to use the data but are not authorized to make them available to third parties. Requests for access to the data are therefore subject to specific authorization by the AEMPS.

## Abbreviations

AOR: Adjusted odds ratio
BIFAP: Base de datos para la Investigación Farmacoepidemiológica en el Ámbito Público
CI: Confidence interval
CMBD: Conjunto Mínimo Básico de Datos
CS: Chondroitin sulfate
NSAID: Nonsteroidal anti-inflammatory drug
OA: Osteoarthritis
OR: Odds ratio
SYSADOA: Symptomatic slow-acting drug for osteoarthritis
