# Supplementary material for: Long-term use of drugs used for knee osteoarthritis and their association with primary arthroplasty: a nested case-control study
Source: Arthroplasty. 2026 Aug 7;8:59. doi: 10.1186/s42836-026-00421-7 (PMC13450516; doi:10.1186/s42836-026-00421-7)
Supplement: Supplementary file 1 [file 42836_2026_421_MOESM1_ESM.docx]

**Supplementary Material**

Long-term use of drugs used for knee osteoarthritis and their association with primary arthroplasty: a nested case-control study

**Fig. S1. Study timeline and exposure assessment.** Patients with a recorded diagnosis of knee osteoarthritis entered the study cohort when they fulfilled eligibility criteria (age 40 to 89 years, with at least 1 year of follow-up with their primary care physician). Patients were excluded if, before the start date, they had a record of cancer, hip, knee, or femur fracture, a major traumatism requiring hospitalization, or any inflammatory arthropathy (excepting gout) and/or had ≥3 months of consecutive prescriptions for any study drug during the year before cohort entry (i.e., prevalent regular users). For cases, index date was defined as 180 days before the record of primary knee arthroplasty (in sensitivity analyses, lag periods of 120 and 365 days were also considered). Drug exposure was assessed from the start date until the index date.

**Fig. S2. Assessment of the potential modification effect by sex and age group (< 70 and ≥ 70 years old) on the association of primary knee arthroplasty with the use of non-opioid analgesics by duration of treatment.** The test of interaction was done comparing the adjusted OR of duration of use > 5 years vs. < 1 year in women with that of men, and the adjusted OR > 5 years vs. < 1 year observed in patients younger than 70 years old with that of patients aged 70 years or older. * Adjusted for age, sex, number of visits to primary care physician during the year prior to index date, obesity, diabetes (recorded as such and/or use of glucose-lowering drugs), dyslipidemia (recorded as such and/or use of lipid-lowering drugs), peripheral artery disease, hypertension, heart failure, atrial fibrillation, angina pectoris (recorded as such and/or use of nitrates), myocardial infarction, ischemic stroke (including non-specified stroke and transient ischemic attack), hemorrhagic stroke, chronic obstructive pulmonary disease, asthma, hyperuricemia, gout, thromboembolic disease, chronic hepatitis, cirrhosis, chronic kidney disease, depression, anxiety, Alzheimer’s disease, dementia, Parkinson disease and use of NSAIDs, SYSADOAs, tramadol in fixed-dose combination with paracetamol, and opioids.

**Fig. S3. Assessment of the potential modification effect by sex and age group (< 70 and ≥ 70 years old) on the association of primary knee arthroplasty with the use of tramadol in fixed-dose combination with paracetamol by duration of treatment.** The test of interaction was done comparing the adjusted OR of duration of use > 5 years vs. < 1 year in women with that of men, and the adjusted OR > 5 years vs. < 1 year observed in patients younger than 70 years old with that of patients aged 70 years or older. * Adjusted for factors shown in the footnote of Fig. S2.

**Fig. S4. Assessment of the potential modification effect by sex and age group (< 70 and ≥ 70 years old) on the association of primary knee arthroplasty with the use of opioids by duration of treatment.** The test of interaction was done comparing the adjusted OR of duration of use > 5 years vs. < 1 year in women with that of men, and the adjusted OR > 5 years vs. < 1 year observed in patients younger than 70 years old with that of patients aged 70 years or older. * Adjusted for factors shown in the footnote of Fig. S2.

**Table S1.** Association of primary knee arthroplasty with the use of NSAIDs by duration of treatment, adjusting for the potential confounding factors at start date.

|  | **Cases** | **Controls** | **Crude OR** | **Adjusted OR*** |
| --- | --- | --- | --- | --- |
|  | ***N* = 15,892** | ***N* = 45,235** | **(95% CI)** | **(95% CI)** |
| Use of NSAIDs | 13,970 (87.9) | 37,755 (83.5) |  |  |
| By duration: |  |  |  |  |
| < 1 year | 8,271 (52.1) | 26,937 (59.6) | 1 (ref) | 1 (ref) |
| 1–3 years | 3,655 (23.0) | 7,582 (16.8) | 1.75 (1.67–1.84) | 1.53 (1.45–1.61) |
| > 3–5 years | 1,249 (7.9) | 2,073 (4.6) | 2.38 (2.21–2.58) | 1.98 (1.82–2.15) |
| > 5 years | 795 (5.0) | 1,163 (2.6) | 2.97 (2.69–3.29) | 2.45 (2.20–2.73) |

*Abbreviations:* CI: confidence interval; OR: odds ratio. *Adjusted for factors shown in the footnote of Fig. S2.

**Table S2.** Association of primary knee arthroplasty with the use of NSAIDs by duration of treatment, adjusting for the follow-up time.

|  | **Cases** | **Controls** | **Crude OR** | **Adjusted OR*** |
| --- | --- | --- | --- | --- |
|  | ***N* = 15,892** | ***N* = 45,235** | **(95% CI)** | **(95% CI)** |
| Use of NSAIDs | 13,970 (87.9) | 37,755 (83.5) |  |  |
| By duration: |  |  |  |  |
| < 1 year | 8,271 (52.1) | 26,937 (59.6) | 1 (ref) | 1 (ref) |
| 1–3 years | 3,655 (23.0) | 7,582 (16.8) | 1.75 (1.67–1.84) | 1.50 (1.43–1.59) |
| > 3–5 years | 1,249 (7.9) | 2,073 (4.6) | 2.38 (2.21–2.58) | 1.91 (1.76–2.09) |
| > 5 years | 795 (5.0) | 1,163 (2.6) | 2.97 (2.69–3.29) | 2.42 (2.17–2.71) |

*Abbreviations:* CI: confidence interval; OR: odds ratio. *Adjusted for factors shown in the footnote of Fig. S2.

**Table S3.** Association of primary knee arthroplasty with the use of NSAIDs by duration of treatment, using a lag time of 120 and 365 days.

| **Lag time of 120 days** | **Cases (%)** | **Controls (%)** | **Crude OR** | **Adjusted OR*** |
| --- | --- | --- | --- | --- |
|  | ***N* = 15,931** | ***N* = 45,462** | **(95% CI)** | **(95% CI)** |
| Use of NSAIDs | 14,057 (88.2) | 38,330 (84.3) |  |  |
| By duration: |  |  |  |  |
| < 1 year | 8,188 (51.4) | 27,023 (59.4) | 1 (ref) | 1 (ref) |
| 1–3 years | 3,761 (23.6) | 7,786 (17.1) | 1.76 (1.68–1.85) | 1.52 (1.44–1.61) |
| > 3–5 years | 1,287 (8.1) | 2,277 (5.0) | 2.24 (2.07–2.41) | 1.81 (1.66–1.97) |
| > 5 years | 821 (5.2) | 1,244 (2.7) | 2.84 (2.57–3.13) | 2.20 (1.97–2.45) |
| **Lag time of 365 days** | **Cases (%)** | **Controls (%)** | **Crude OR** | **Adjusted OR*** |
|  | ***N* = 15,604** | ***N* = 43,967** | **(95% CI)** | **(95% CI)** |
| Use of NSAIDs | 13,500 (86.5) | 36,346 (82.7) |  |  |
| By duration: |  |  |  |  |
| < 1 year | 8,292 (53.1) | 26,099 (59.4) | 1 (ref) | 1 (ref) |
| 1–3 years | 3,389 (21.7) | 7,262 (16.5) | 1.65 (1.57–1.74) | 1.42 (1.31–1.53) |
| > 3–5 years | 1,115 (7.2) | 1,989 (4.5) | 2.15 (1.98–2.33) | 1.66 (1.46–1.88) |
| > 5 years | 704 (4.5) | 996 (2.3) | 2.92 (2.63–3.25) | 2.36 (2.00–2.79) |

*Abbreviations:* CI: confidence interval; OR: odds ratio.

*Note*: For each lag-time definition, case-control matching was performed independently. Therefore, the number of cases may differ across analyses because changes in the lag period affect control eligibility, leading to the exclusion of some cases with no matched controls and the inclusion of others that met matching criteria under the alternative lag definition.

*Adjusted for factors shown in the footnote of Fig. S2.

**Table S4.** Association of primary knee arthroplasty with the use of SYSADOAs by duration of treatment, adjusting for the potential confounding factors at start date.

|  | **Cases** | **Controls** | **Crude OR** | **Adjusted OR*** |
| --- | --- | --- | --- | --- |
|  | ***N* = 15,892** | ***N* = 45,235** | **(95% CI)** | **(95% CI)** |
| Use of SYSADOAs | 5,283 (33.2) | 11,666 (25.8) |  |  |
| By duration: |  |  |  |  |
| < 1 year | 3,291 (20.7) | 7,430 (16.4) | 1 (ref.) | 1 (ref.) |
| 1–3 years | 1,314 (8.3) | 2,813 (6.2) | 1.08 (1.00–1.17) | 1.03 (0.95–1.12) |
| > 3–5 years | 447 (2.8) | 967 (2.1) | 1.10 (0.98–1.24) | 0.99 (0.87–1.13) |
| > 5 years | 231 (1.5) | 456 (1.0) | 1.25 (1.06–1.48) | 1.11 (0.93–1.53) |

*Abbreviations:* CI: confidence interval; OR: odds ratio. *Adjusted for factors shown in the footnote of Fig. S2.

**Table S5.** Association of primary knee arthroplasty with the use of SYSADOAs by duration of treatment, adjusting for the follow-up time.

|  | **Cases** | **Controls** | **Crude OR** | **Adjusted OR*** |
| --- | --- | --- | --- | --- |
|  | ***N* = 15,892** | ***N* = 45,235** | **(95% CI)** | **(95% CI)** |
| Use of SYSADOAs | 5,283 (33.2) | 11,666 (25.8) |  |  |
| By duration: |  |  |  |  |
| < 1 year | 3,291 (20.7) | 7,430 (16.4) | 1 (ref.) | 1 (ref.) |
| 1–3 years | 1,314 (8.3) | 2,813 (6.2) | 1.08 (1.00–1.17) | 1.03 (0.94–1.12) |
| > 3–5 years | 447 (2.8) | 967 (2.1) | 1.10 (0.98–1.24) | 1.01 (0.89–1.16) |
| > 5 years | 231 (1.5) | 456 (1.0) | 1.25 (1.06–1.48) | 1.13 (0.94–1.36) |

*Abbreviations:* CI: confidence interval; OR: odds ratio. *Adjusted for factors shown in the footnote of Fig. S2.

**Table S6.** Association of primary knee arthroplasty with the use of SYSADOAs by duration of treatment, using a lag time of 120 and 365 days.

| **Lag time of 120 days** | **Cases (%)** | **Controls (%)** | **Crude OR** | **Adjusted OR*** |
| --- | --- | --- | --- | --- |
|  | ***N* = 15,931** | ***N* = 45,462** | **(95% CI)** | **(95% CI)** |
| Use of SYSADOAs | 5,327 (33.4) | 11,989 (26.4) |  |  |
| By duration: |  |  |  |  |
| < 1 year | 3,292 (20.7) | 7,652 (16.8) | 1 (ref) | 1 (ref) |
| 1–3 years | 1,338 (8.4) | 2,837 (6.2) | 1.12 (1.03–1.21) | 1.06 (0.98–1.16) |
| > 3–5 years | 453 (2.8) | 956 (2.1) | 1.14 (1.01–1.29) | 1.07 (0.93–1.22) |
| > 5 years | 244 (1.5) | 544 (1.2) | 1.14 (0.97–1.33) | 1.05 (0.88–1.25) |
| **Lag time of 365 days** | **Cases (%)** | **Controls (%)** | **Crude OR** | **Adjusted OR*** |
|  | **N = 15,604** | **N = 43,967** | **(95% CI)** | **(95% CI)** |
| Use of SYSADOAs | 4,977 (31.9) | 11,059 (25.2) |  |  |
| By duration: |  |  |  |  |
| < 1 year | 3,120 (20.0) | 7,276 (16.6) | 1 (ref) | 1 (ref) |
| 1–3 years | 1,248 (8.0) | 2,526 (5.8) | 1.19 (1.10–1.30) | 1.05 (0.93–1.20) |
| > 3–5 years | 404 (2.6) | 862 (2.0) | 1.17 (1.03–1.32) | 0.99 (0.82–1.19) |
| > 5 years | 205 (1.3) | 395 (0.90) | 1.33 (1.11–1.58) | 1.36 (1.03–1.78) |

*Abbreviations:* CI: confidence interval; OR: odds ratio.

*Note*: For each lag-time definition, case-control matching was performed independently. Therefore, the number of cases may differ across analyses because changes in the lag period affect control eligibility, leading to the exclusion of some cases with no matched controls and the inclusion of others that met matching criteria under the alternative lag definition.

*Adjusted for factors shown in the footnote of Fig. S2.

**Table S7**. Risk of knee arthroplasty associated with the duration of treatment of paracetamol and metamizole.

|  | **Cases** | **Controls** | **Crude OR** | **Adjusted OR*** |
| --- | --- | --- | --- | --- |
|  | ***N* = 15,892** | ***N* = 45,235** | **(95% CI)** | **(95% CI)** |
| Use of paracetamol | 12,772 (80.4) | 35,155 (77.7) |  |  |
| By duration: |  |  |  |  |
| < 1 year | 7,576 (47.7) | 22,237 (49.2) | 1 (ref) | 1 (ref) |
| 1–3 years | 3,432 (21.6) | 8,403 (18.6) | 1.25 (1.19–1.31) | 1.02 (0.96–1.07) |
| > 3–5 years | 1,199 (7.5) | 3,063 (7.9) | 1.24 (1.15–1.34) | 0.90 (0.83–0.97) |
| > 5 years | 565 (3.6) | 1,452 (3.2) | 1.26 (1.14–1.40) | 0.90 (0.80–1.01) |
| Use of metamizole | 9,681 (60.9) | 23,850 (52.7) |  |  |
| By duration: |  |  |  |  |
| < 1 year | 7,457 (46.9) | 19,381 (42.9) | 1 (ref) | 1 (ref) |
| 1–3 years | 1,625 (10.2) | 3,201 (7.1) | 1.38 (1.29–1.47) | 1.08 (1.01–1.17) |
| > 3–5 years | 456 (2.9) | 931 (2.1) | 1.37 (1.22–1.54) | 0.97 (0.85–1.10) |
| > 5 years | 143 (0.90) | 337 (0.74) | 1.22 (1.00–1.48) | 0.87 (0.70–1.08) |

*Abbreviations:* CI: confidence interval; OR: odds ratio. *Adjusted for factors shown in the footnote of Fig. S2.

**Table S8.** Association of primary knee arthroplasty with the use of non-opioid analgesics by duration of treatment, using a lag time of 120 and 365 days.

| **Lag time of 120 days** | **Cases (%)** | **Controls (%)** | **Crude OR** | **Adjusted OR*** |
| --- | --- | --- | --- | --- |
|  | ***N* = 15,931** | ***N* = 45,462** | **(95% CI)** | **(95% CI)** |
| Use of non-opioid analgesics | 14,240 (89.4) | 39,147 (86.1) |  |  |
| By duration: |  |  |  |  |
| < 1 year | 7,889 (49.5) | 23,850 (52.5) | 1 (ref) | 1 (ref) |
| 1–3 years | 4,084 (25.6) | 9,779 (21.5) | 1.34 (1.28–1.40) | 1.06 (1.00–1.11) |
| > 3–5 years | 1,537 (9.7) | 3,665 (8.1) | 1.40 (1.31–1.50) | 0.97 (0.90–1.05) |
| > 5 years | 730 (4.6) | 1,853 (4.1) | 1.36 (1.23–1.49) | 0.92 (0.83–1.02) |
| **Lag time of 365 days** | **Cases (%)** | **Controls (%)** | **Crude OR** | **Adjusted OR*** |
|  | ***N* = 15,604** | ***N* = 43,967** | **(95% CI)** | **(95% CI)** |
| Use of non-opioid analgesics | 13,591 (87.1) | 37,068 (84.3) |  |  |
| By duration: |  |  |  |  |
| < 1 year | 8,065 (51.7) | 23,546 (53.6) | 1 (ref) | 1 (ref) |
| 1–3 years | 3,679 (23.6) | 8,900 (20.2) | 1.29 (1.23–1.35) | 0.95 (0.89–1.02) |
| > 3–5 years | 1,289 (8.3) | 3,173 (7.2) | 1.31 (1.22–1.41) | 0.82 (0.74–0.92) |
| > 5 years | 558 (3.6) | 1449 (3.3) | 1.28 (1.15–1.42) | 0.84 (0.72–0.99) |

*Abbreviations:* CI: confidence interval; OR: odds ratio.

*Note*: For each lag-time definition, case-control matching was performed independently. Therefore, the number of cases may differ across analyses because changes in the lag period affect control eligibility, leading to the exclusion of some cases with no matched controls and the inclusion of others that met matching criteria under the alternative lag definition.

*Adjusted for factors shown in the footnote of Fig. S2.

**Table S9.** Association of primary knee arthroplasty with the use of tramadol and paracetamol by duration of treatment, using a lag time of 120 and 365 days.

| **Lag time of 120 days** | **Cases (%)** | **Controls (%)** | **Crude OR** | **Adjusted OR*** |
| --- | --- | --- | --- | --- |
|  | ***N* = 15,931** | ***N* = 45462** | **(95% CI)** | **(95% CI)** |
| Users of tramadol+paracetamol | 8,697 (54.6) | 17,779 (39.1) |  |  |
| By duration: |  |  |  |  |
| < 1 year | 6,395 (40.1) | 13,996 (30.8) | 1 (ref) | 1 (ref) |
| 1–3 years | 1,630 (10.2) | 2,630 (5.8) | 1.40 (1.31–1.50) | 1.28 (1.19–1.38) |
| > 3–5 years | 482 (3.0) | 792 (1.7) | 1.42 (1.26–1.60) | 1.26 (1.11–1.44) |
| > 5 years | 190 (1.2) | 361 (0.79) | 1.24 (1.03–1.48) | 1.08 (0.88–1.31) |
| **Lag time of 365 days** | **Cases (%)** | **Controls (%)** | **Crude OR** | **Adjusted OR*** |
|  | ***N* = 15,604** | ***N* = 43967** | **(95% CI)** | **(95% CI)** |
| Users of tramadol+paracetamol | 7,603 (48.7) | 15,918 (36.2) |  |  |
| By duration: |  |  |  |  |
| < 1 year | 5,737 (36.8) | 12,684 (28.9) | 1 (ref) | 1 (ref) |
| 1–3 years | 1,346 (8.6) | 2,268 (5.2) | 1.37 (1.27–1.48) | 1.13 (1.01–1.27) |
| > 3–5 years | 388 (2.5) | 689 (1.6) | 1.34 (1.18–1.52) | 1.02 (0.84–1.24) |
| > 5 years | 132 (0.85) | 277 (0.63) | 1.14 (0.92–1.40) | 0.96 (0.69–1.35) |

*Abbreviations:* CI: confidence interval; OR: odds ratio.

*Note*: For each lag-time definition, case-control matching was performed independently. Therefore, the number of cases may differ across analyses because changes in the lag period affect control eligibility, leading to the exclusion of some cases with no matched controls and the inclusion of others that met matching criteria under the alternative lag definition.

*Adjusted for factors shown in the footnote of Fig. S2.

**Table S10.** Association of primary knee arthroplasty with the use of opioids by duration of treatment, using a lag time of 120 and 365 days.

| *Lag time of 120 days* | **Cases (%)** | **Controls (%)** | **Crude OR** | **Adjusted OR*** |
| --- | --- | --- | --- | --- |
|  | ***N* = 15,931** | ***N* = 45,462** | **(95%CI)** | **(95%CI)** |
| Use of opioids | 4,388 (27.5) | 8,225 (18.1) |  |  |
| By duration: |  |  |  |  |
| < 1 year | 3,037 (19.1) | 6,179 (13.6) | 1 (ref) | 1 (ref) |
| 1–3 years | 867 (5.4) | 1,253 (2.8) | 1.43 (1.30–1.57) | 1.19 (1.07–1.33) |
| > 3–5 years | 308 (1.9) | 500 (1.1) | 1.30 (1.12–1.51) | 1.02 (0.86–1.20) |
| > 5 years | 176 (1.1) | 293 (0.64) | 1.31 (1.08–1.59) | 0.93 (0.75–1.16) |
| *Lag time of 365 days* | **Cases (%)** | **Controls (%)** | **Crude OR** | **Adjusted OR*** |
|  | ***N* = 15,604** | ***N* = 43,967** | **(95%CI)** | **(95%CI)** |
| Use of opioids | 3,730 (23.9) | 7,326 (16.7) |  |  |
| By duration: |  |  |  |  |
| < 1 year | 2,637 (16.9) | 5,592 (12.7) | 1 (ref) | 1 (ref) |
| 1–3 years | 709 (4.5) | 1,109 (2.5) | 1.37 (1.23–1.52) | 1.29 (1.10–1.52) |
| > 3–5 years | 250 (1.6) | 387 (0.88) | 1.40 (1.19–1.66) | 1.17 (0.91–1.51) |
| > 5 years | 134 (0.86) | 238 (0.54) | 1.27 (1.02–1.58) | 1.34 (0.95–1.87) |

*Abbreviations:* CI: confidence interval; OR: odds ratio.

*Note*: For each lag-time definition, case-control matching was performed independently. Therefore, the number of cases may differ across analyses because changes in the lag period affect control eligibility, leading to the exclusion of some cases with no matched controls and the inclusion of others that met matching criteria under the alternative lag definition.

*Adjusted for factors shown in the footnote of Fig. S2.
